# Supplementary material for: Experiences of integrating a psychological intervention into a youth-led empowerment program targeting out-of-school adolescents, in urban informal settlements in Kenya: A qualitative study
Source: PLoS One. 2024 Apr 3;19(4):e0300463. doi: 10.1371/journal.pone.0300463 (PMC10990221; doi:10.1371/journal.pone.0300463)
Supplement: S1 Table — (DOCX) [file pone.0300463.s001.docx]

**S1 Table: Socio-demographics of Respondents Interviewed**

| **Variables** | **Adolescents Boys  (N=22)** | **Adolescents Girls  (N=22)** | **Parents  (N=19)** | **Mentors  (N=17)** | **Stakeholders  (N=11)** | **Partners (N=7)** | **Total  (N=98)** |
| --- | --- | --- | --- | --- | --- | --- | --- |
| **Sex** |  |  |  |  |  |  |  |
| Male | 22 (22.4%) | 0 (0%) | 5 (5.1%) | 5%5.1%) | 7 (7.1%) | 3 (3.1%) | 42 (42.9%) |
| Female | 0 (0%) | 22 (22.4%) | 14 (14.1%) | 12(12.2%) | 4 (4.1%) | 4 (4.1%) | 56 (57.1%) |
| Total | 22 (22.4%) | 22 (22.4%) | 19 (19.4%) | 17(17.3%) | 11(11.2%) | 7(7.2%) | 98 (100%) |
| **Age (mean)** | 18.45 | 18.59 | 43.15 | 31.11 | 48.63 | 22.71 | n/a |
| **Highest Education** |  |  |  |  |  |  |  |
| No education | 0 (0%) | 0 (0%) | 0 (0%) | 0 (0%) | 0 (0%) | 0 (0%) | 0 (0%) |
| Incomplete primary | 6 (6.1%) | 1 (1.0%) | 5 (5.1%) | 0 (0%) | 0 (0%) | 0 (0%) | 12 (12.2%) |
| Primary | 3 (3.1%) | 10 (10.2%) | 3 (3.1%) | 0 (0%) | 1 (1.0%) | 0 (0%) | 17 (17.3%) |
| Incomplete Secondary | 12 (12.2%) | 9 (9.2%) | 5 (5.1%) | 0 (0%) | 1 (1.0%) | 4 (0%) | 31 (31.6%) |
| Secondary | 1 (1.0%) | 2 (2.0%) | 3 (3.1%) | 5(5.1%) | 4 (4.1%) | 1 (0%) | 16 (16.3) |
| Higher | 0 (0%) | 0 (0%) | 3 (3.1%) | 12(12.2%) | 5 (5.1%) | 2 (0%) | 22 (22.4) |
| **Marital Status** |  |  |  |  |  |  |  |
| Never Married | 18 (18.4%) | 19 (19.4%) | 1 (2.4%) | 2 (2.0%) | 0 (0%) | 3 (3.1%) | 49 (50.1%) |
| Married | 2 (2.0%) | 2 (2.0%) | 8 (19.1%) | 11 (11.2%) | 10 (10.2%) | 4 (4.1%) | 37 (37.8%) |
| Widowed | 0 (0%) | 0 (0%) | 3 (7.1%) | 2 (2.0%) | 0 (0%) | 0 (0%) | 3 (3.1%) |
| Divorced | 0 (0%) | 0 (0%) | 0 (0%) | 2 (2.0%) | 0 (0%) | 0 (0%) | 3 (3.1%) |
| Separated | 2 (2.0%) | 1 (1.0%) | 0 (0%) | 2 (2.0%) | 1 (0%) | 0 (0%) | 6 (6.1%) |
| **Religion** |  |  |  |  |  |  |  |
| Muslim | 4 (4.1%) | 0 (0%) | 0 (0%) | 0 (0%) | 2 (2.0%) | 0 (0%) | 6 (6.1%) |
| Catholic | 8 (8.2%) | 6 (6.1%) | 4 (4.1%) | 4 (4.1%) | 4 (4.1%) | 1 (9.5%) | 27 (27.6%) |
| Protestant | 7 (7.1%) | 15 (15.3%) | 12 (12.2%) | 12 (12.2%) | 4 (4.1%) | 5 (0%) | 55 (55.1%) |
| Other | 3 (3.1%) | 1 (1.0%) | 3 (3.1%) | 1 (1.0%) | 1 (1.0%) | 1 (0%) | 10 (10.2%) |
| **Occupation** |  |  |  |  |  |  |  |
| CHV’s/HTS | 0 (0%) | 0 (0%) | 2 (2.0%) | 6 (6.1%) | 2 (2.0%) | 0 (0%) | 10 (10.2%) |
| Casual Laborers | 13 (13.3%) | 4 (4.1%) | 6 (6.1%) | 0 (0%) | 0 (0%) | 2 (0%) | 25 (25.5%) |
| Employed (not specified) | 0 (0%) | 0 (0%) | 3 (3.1%) | 3 (3.1%) | 4 (4.1%) | 0 (0%) | 10 (10.2%) |
| Teacher | 0 (0%) | 0 (0%) | 0 (0%) | 1 (1.0%) | 1 (1.0%) | 0 (0%) | 2 (2.0%) |
| Security guard | 0 (0%) | 0 (0%) | 3 (3.1%) | 0 (0%) | 0 (0%) | 0 (0%) | 4 (4.1%) |
| Paralegal | 0 (0%) | 0 (0%) | 0 (0%) | 1 (1.0%) | 2 (2.0%) | 0 (0%) | 2 (2.0%) |
| Radio presenter | 0 (0%) | 0 (0%) | 0 (0%) | 1 (1.0%) | 0 (0%) | 0 (0%) | 1 (1.0%) |
| Clinical Officer | 0 (0%) | 0 (0%) | 0 (0%) | 0 (0%) | 1 (1.0%) | 0 (0%) | 1 (1.0%) |
| Hair dresser | 0 (0%) | 2 (2.0%) | 0 (0%) | 0 (0%) | 0 (0%) | 0 (0%) | 2 (2.0%) |
| Own business | 2 (2.0%) | 5 (5.1%) | 6 (6.1%) | 3 (3.1%) | 1 (1.0%) | 4 (4.1%) | 21 (21.4%) |
| Unemployed | 4 (4.1%) | 9 (9.2%) | 1 (1.0%) | 0 (0%) | 0 (0%) | 1 (1.0%) | 15 (15.3%) |
| Mentor | 0 (0%) | 0 (0.0%) | 0 (0%) | 4 (4.1%) | 0 (0%) | 0 (0%) | 4 (4.1%) |
| Student | 0 (0%) | 0 (0%) | 0 (0%) | 0 (0%) | 0 (0%) | 0 (0%) | 4 (4.1%) |
| Other | 2 (2.0%) | 2 (2.0%) | 1 (1.0%) | 1 (1.0%) | 4 (4.0%) | 0 (0%) | 9 (9.2%) |
| Painter/ Artist | 1 (1.0%) | 0 (0%) | 0 (0%) | 0 (0%) | 0 (0%) | 0 (0%) | 1 (1.0%) |
| Village elder | 0 (0%) | 0 (0%) | 0 (0%) | 0 (0%) | 2 (2.0%) | 0 (0%) | 2 (2.0%) |
| Pastor | 0 (0%) | 0 (0%) | 0 (0%) | 0 (0%) | 2 (2.0%) | 0 (0%) | 2 (2.0%) |
| **Living arrangement** |  |  |  |  |  |  |  |
| Both Parents | 3 (6.8%) | 6 (13.6%) | 0 (0%) | 0 (0%) | 0 (0%) | 0 (0%) | 9 (20.5%) |
| Mother only | 7 (15.9%) | 6 (13.6%) | 0 (0%) | 0 (0%) | 0 (0%) | 0 (0%) | 13 (29.5) |
| Father only | 0 (0%) | 1 (2.3%) | 0 (0%) | 0 (0%) | 0 (0%) | 0 (0%) | 1 (2.3%) |
| Spouse | 3 (6.8%) | 3 (6.8%) | 0 (0%) | 0 (0%) | 0 (0%) | 0 (0%) | 6 (13.6%) |
| Other immediate relatives | 2 (4.5%) | 5 (11.4%) | 0 (0%) | 0 (0%) | 0 (0%) | 0 (0%) | 7 (15.9) |
| Distant relatives | 4 (9.1%) | 1 (2.3%) | 0 (0%) | 0 (0%) | 0 (0%) | 0 (0%) | 5 (11.4%) |
| Alone | 3 (6.8%) | 0 (0%) | 0 (0%) | 0 (0%) | 0 (0%) | 0 (0%) | 3 (6.8%) |
| **Location** |  |  |  |  |  |  |  |
| Kariobangi | 13 (13.3%) | 11 (11.2%) | 7 (7.1%) | 7 (7.1%) | 6 (6.1%) | 5 (5.1%) | 49 (50%) |
| Rhonda | 9 (9.2%) | 11 (11.2%) | 12 (12.2%) | 10 (10.2%) | 5 (5.1%) | 2 (2.0%) | 49 (50%) |
