# Supplementary material for: Experiences of integrating a psychological intervention into a youth-led empowerment program targeting out-of-school adolescents, in urban informal settlements in Kenya: A qualitative study
Source: PLoS One. 2024 Apr 3;19(4):e0300463. doi: 10.1371/journal.pone.0300463 (PMC10990221; doi:10.1371/journal.pone.0300463)
Supplement: S2 Table — (DOCX) [file pone.0300463.s002.docx]

| **S2 Table: Coding Matrix** | | |
| --- | --- | --- |
| **Code** | **Sub-code(s)** | **Definition** |
| **1.0 GENERAL BTG PROGRAM** | 1.1 Knowledge on BTG  1.1.1 What BTG offers  1.2 Likes  1.3 Dislikes  1.4 Areas for improvement | Used to capture comments on what respondents know about the BTG program in general, what it offers, their likes, dislikes and recommendations on what should be done differently. |
| - 1. **MENTOR TRAINING SESSIONS** | - 1. Meeting description   2. Topics   3. Likes   4. Areas of improvement   2.5 Impact  2.5.1. Mentors skilled in delivering group and individual content  2.5.1.1 Preparation for BTG program | Used to capture comments on the understanding of the trainings offered, what the mentors liked about the training, and any areas of improvement.  It also captures comments on the knowledge and skills acquired, and how the trainings prepared the mentors for the BTG program.  .  Note/ Comments from mentors |
| **3.0 SAFE SPACE MEETINGS** | 3.1 General Meeting overview  3.1.1 Activities  3.1.2 Likes  3.1.3 Dislikes  3.1.4 Similar meetings attended  3.1.4.1 Observed differences | Used to capture comments on safe space meetings, the typical activities held, and the likes and dislikes.  Also captures comments on any similar meetings attended and any perceived differences. |
|  | 3.2 Facilitation  3.2.1 Likes  3.2.1.1 General sessions  3.2.1.2 MH sessions  3.2.2 Dislikes  3.2.3 Similar programs  3.2.3.1 Observed differences | Used to capture comments on what mentors liked and disliked about facilitating the general group meetings and MH sessions in particular.  Also captures comments on any similar meetings facilitated and any perceived differences. |
|  | 3.3 Meetings Impact  3.3.1 Topics  3.3.2 Skills & knowledge acquired  3.3.3 Proposed/suggested additional topics  3.3.3.1 Adolescents  3.3.3.2 Mentors  3.3.4 Behavior change  3.3.4.1 Adolescent  3.3.4.2 Child/partner relationship  3.3.5 Strong sense of social support and connectedness | Used to capture comments on topics discussed, the skills and knowledge impacted, any proposed additional topics missing in BTG program  Also captures comments on the how the life of the adolescents has changed and also how the adolescents and partner or parent relationship has changed.  Captures comments on how the participants feel about the improved sense of social support and connectedness since joining the program.  NB. Green highlight requires clarification |
|  | 3.4 Support structures  3.4.1 Improvised structures  3.4.1.1 Effectiveness  3.4.2 Recommendations | Used to capture comments on any support structures mentors put in place to improve meeting attendance and effectiveness of the structures in improving the attendance.  Also captures any recommendations for structures that would enhance the group session attendance. |
|  | 3.5 Mentor Interactions  3.5.1 Experiences  3.5.2 Roles and Responsibilities  3.5.3 Likes  3.5.4 Dislikes  3.5.5 Areas of improvement | Used to capture comments on the respondent’s interaction with the mentor, their roles and responsibilities, what they liked or disliked about the mentor and suggested areas of improvement. |
|  | 3.6 Attendance  3.6.1 Reasons for absenteeism  3.6.1.1 Work  3.6.1.2 House chores  3.6.1.3 Lack of motivation/incentives  3.6.2 Suggested incentives | Used to capture comments on safe space meetings attendance, the reason why the respondents’ missed meetings, and any non-financial incentives that would motivate meeting attendance. |
|  | 3.7 Mental Health Discussions  3.7.1 Understanding of MI  3.7.1.1 Change in MI understanding | Used to capture comments on respondents understanding of mental illness and how this understanding has changed since joining the program.  Also captures comments on change in how they view causes, signs and symptoms or treatment of MH. |
|  | 3.8 Increased comfort discussing and seeking out resources for mental health  3.8.2 Discussions  3.8.2.1 Adolescent  3.8.2.2 Mentor/mentee  3.8.2.3 Parent/Partner | Captures comments on improved ease of discussing MH issues and seeking out resources for the same.  Also captures comments on the changes in who the adolescents discuss their MH challenges with, mentor mentee discussions on MH and mentee-partner/ parent MH discussions |
|  | 3.9 Mentee response  3.9.1 Reasons for reaction  3.10 Satisfaction  3.11 Skills gained  3.12 Areas of improvement  3.12.1 General meetings  3.12.2 MH sessions | Used to capture comments on how the mentees reacted to MH discussion and perceived reasons for reactions.  Also captures comments on skills and knowledge gained, the level of satisfaction and areas of improvement of the general meetings and MH sessions. |
|  | 3.13 MH Linkages  3.13.1 Areas of life  3.13.2 Life situations  3.14 Mentor experience | Captures comments on understanding of MH influence in different areas of life and how the mentors were able to explain the linkage between MH and different areas of life.  Also captures comments on respondents understanding of different situations in life that affect MH. |
|  | 3.15 Balance with other topics | Captures comments on how mental health discussions were done in relation with other topics and how the discussions can be balanced with other topics. |
|  | 3.16 Community mobilization/awareness  3.16.1 Likes  3.16.2 Discussion topics  3.16.3 Areas of improvement  3.16.3.1 Mental Health  3.16.3.2 Community meetings  3.16.4 Additional topics | Used to capture comments on the respondent’s knowledge on community awareness activities that is the experience, the likes, the knowledge on what was discussed, the areas of improvement and additional topics to be added in the discussions. |
| **4.0 ONE ON ONE SESSIONS** | 4.1 Session overview  4.1.1 Awareness  4.1.1.1 Source of information  4.1.2 Experience  4.1.3 Satisfaction/Likes  4.1.4 Areas of improvement  4.1.5 Discussions  4.1.5.1 Parents  4.1.5.2 Partner  4.1.5.3 Peers  4.1.6 Perception  4.1.6.1 Parents  4.1.6.2 Partner  4.1.6.3 Peers  4.1.7 Non attendance  4.1.7.1 Reasons  4.1.7.2 Perceived/ Imagined benefits | Used to capture comments on the respondents’ knowledge about the one on one sessions, their source of information about the one on one session, their experience, the likes and areas of improvement.  It also captures comments on who the adolescents discuss with their attendance of the session and their perceptions. Also captures comments on the adolescents who never attended any session, their reasons and the benefits they imagine they would have gained from attending. |
| **Mentor** | 4.2 Mentor experience  4.2.1 Mentee identification  4.2.2 Information process  4.2.3Likes  4.2.4 Challenges  4.2.4.1 Interventions  4.2.5 Benefits  4.2.5.1 Knowledge and skills impacted  4.2.5.2 Impact on adolescent lives  4.2.6 Perception  4.2.6.1 Parents  4.2.6.2 Partner  4.2.6.3 Peers  4.2.7 One on one not offered  4.2.7.1 Reasons  4.2.7.2 Potential benefits | Used to capture comments related to mentors’ experience with one on one sessions, if they ever conducted one on one session, how they identified the mentees, their experience, what they liked about the session, the challenges they experienced, the changes they would want to see on the sessions, what the mentees learnt and how the lives of the mentees changed from attending the sessions.  Also used to capture the views on the mentors on the perception of parents, partner, and peers about the one on one sessions. |
| **5.0 REFERRAL EXPERIENCE** | 5.1 General experience  5.1.1 Mentee Referred  5.1.1.1 Yes  5.1.1.2 No  5.1.2Mentor  5.1.2.1 Yes  5.1.2.2 No  5.1.3 Adolescent experience  5.1.3.1 Provision of information  5.1.3.2 To whom Referred  5.1.4 Benefits  5.1.5 Challenges  5.1.6 Discussion  5.1.7 Perception  5.1.7.1 Parent perception  5.1.7.2 Partner perception  5.1.7.3 Friend perception  5.1.7.4 Others  5.1.8 Reasons for non-compliance | Used to capture comments related to referral whether one has been referred and description of their referral experience. Captures comments related to how referral information was given to mentee, parent or partner.  Also captures comments on where one was referred and any reasons for not going for the referral. Whom they discussed with the referral and their perceptions about the referral.  Also used to capture comments related to referral services, that is the challenges in access and the perceived benefits, |
| **Mentors** | 5.2 Mentor experience  5.2.1 Referral reasons  5.2.2 Reasons for non-compliance  5.2.3 Information provided  5.2.4 Perceptions  5.2.4.1 Parent  5.2.4.2 Partner  5.2.4.3 Friends/Peers  5.2.5 Benefits  5.2.6 Challenges  5.2.6.1 Improvised Interventions  5.2.7 Recommended interventions | Used to capture comments related to mentor’s experience with referral process, reasons for referral, information provided to the mentees on referral, reasons for non-compliance from the mentee, the challenges faced during referral, how challenges were overcome by the mentors, and the perceptions of the parents, partner and peers on the referral.  It also captures comments on interventions that would improve the referral process. |
